# Preparation, assessment, and swelling study of amphiphilic acrylic acid/chitosan-based semi-interpenetrating hydrogels

**DOI:** 10.3906/kim-2109-50

**Published:** 2021-12-10

**Authors:** Mohammad Reza JOZAGHKAR, Amir SEPEHRIAN AZAR, Farshid ZIAEE, Fakhrosadat MIRTALEB

**Affiliations:** 1 Department of Polymer Science, Iran Polymer and Petrochemical Institute, Tehran, Iran; 2 Department of Chemistry, Ahar Branch, Islamic Azad University, Ahar, Iran

**Keywords:** Amphiphilic hydrogel, chitosan, acrylic acid, swelling behavior, salinity

## Abstract

The aim of this study was to synthesize amphiphilic semi-IPN hydrogels based on acrylic acid (AA) and chitosan (CS) using AIBN as an initiator and N,N’methylene bis acrylamide as a crosslinking agent. The swelling behavior of the hydrogels was evaluated at a variety of pH values, temperature, the salinity of media and time, and the swelling mechanism was investigated using Fickian diffusion and Schott’s 2nd-order-kinetics models. FTIR spectroscopy was used to confirm the synthesis of AA/CS hydrogel. The swelling results showed that, in the acidic media, as the CS content increase the hydrogel swelling ratio reduces. It was found that the maximum swelling ratio (8550%) is attained for the sample HAC2 with an AA/CS ratio of 1:0.001 in the alkaline Ph value. It also revealed that the absorption capacity is directly dependent on the temperature and inversely related to the salt concentration. Besides, the absorption capacity of the synthesized hydrogels in the saline solution of Na_2_SO_4_ was higher than NaCl.

## 1. Introduction

In the last few decades, acrylic acid-chitosan amphiphilic superabsorbent hydrogels have received great research interest because these hydrogels can uptake a large amount of water and have antibacterial activities [1, 2]. Due to their excellent properties, chitosan-acrylic acid hydrogels play a key role in wastewater treatment [3, 4].

Chitosan has been known as a renewable biopolymer with considerable biocompatibility, biodegradability, antimicrobial activity, etc. Its properties can be altered under mild conditions due to its reactive amino and hydroxyl group [5]. However, the swelling ratio of chitosan is high just at a low pH value. Introducing poly(acrylic acid) is a potential role to amend the swelling degree of chitosan in a variety of pH values [6].

Ge et al. [7] synthesized the superabsorbent polymer based on chitosan-acrylic acid using the thermal reaction. Shim and coworkers [8] prepared Gamma irradiated poly(acrylic acid)-chitosan hydrogels for the purpose of increasing the drug release aptitude. They reported that release behavior of the drug, 5-fluorouacil from the hydrogel was different based on the pH value of the medium, monomer percentage, and the radiation dose. Zheng et al. [9] investigated the recovery of a valuable metal Ni^2+^ using aqueous dispersion polymerized chitosan-acrylic acid hydrogel. They observed that the prepared absorbent had a good affinity to Ni^2+^. Also, it is observed that chelation interaction among the carboxylated group, and Ni^2+^ is the main mechanism of absorption.

Today, numerous kinds of hydrogels have been formed. Among them, amphiphilic semi-interpenetrating networks (semi-IPN) have been extensively favored because of their excellent properties [10–12]. Marjub et al. [13] used Acrylic acid-chitosan semi-IPN hydrogel for copper (II) and lead (II) ions adsorption from waste water. Torrado and coworkers [14] investigated the drug release behavior of PAAc-chitosan hydrogels. In semi-IPN hydrogels, each polymer network retains its individual characteristics like its homopolymer as well as, when one portion shrinks or swells, another portion could be created for supporting via repulsive and attractive interaction of the whole network. It is noteworthy that when an amphiphilic semi-IPN hydrogel swells, the network can constitute hydrophobic interaction, suggesting the reduction of the swelling degree of the hydrophilic network [15–17].

The aim of this study was to synthesize amphiphilic semi-IPN hydrogels based on acrylic acid and chitosan and characterize their swelling behavior. For this purpose, free radical polymerization was performed using AIBN as a free radical initiator and N,N’methylene bis acrylamide as a crosslinking agent. The effect of chitosan content, pH value, temperature, time, and salinity of media on the swelling properties of prepared hydrogels was evaluated. Moreover, the kinetics and diffusion studies were also carried out.

## 2. Experimental

### 2.1. Materials

All the chemical reagents were supplied from Aldrich Co. (Germany). Chitosan (CS) and acrylic acid (AA) was used as monomers. Azoisobutyronitrile (AIBN, 99%), N,N’methylene bis acrylamide (NNMBA, 99%), sodium chloride (NaCl, 99.5 %), and sodium hydroxide (NaOH, 98%) were used as received.

### 2.2. Synthesis of hydrogels

At first, AA solution was produced via addition of AA (1 mL) to deionized water (1 mL). Then, CS with different amounts of (0.360, 1.82, 3.64, 7.28, 10.94 gr) was added to AA solution and dissolved using ultrasonic for 30 min. After that, 0.0005 g of AIBN and 0.0005 g of NNMBA were added to the solution. The mixtures were injected into the tubes and put down into a bath of hot water at 75 °C for 3 h. After removing the samples from the tube, the hydrogels were cut to the same portion. After that, the samples were weighed and put into distilled water for 24 h. The samples were weighed again and oven-dried at 50°C for 24 h. The designation and composition of the synthesized samples are listed in Table 1.

### 2.3 characterization

The FTIR spectra of the dry semi-IPN hydrogel were scanned using Perkin–Elmer spectrophotometer. The synthesized sample was dried overnight under a vacuum condition, until constant weight. After that, the sample was ground into a fine powder and mixed with KBr powder. The analysis was performed on the wavenumber range from 4000 to 500 cm^−1^.

### 2.4. Swelling studies

The swelling ratio of the synthesized hydrogels was measured in deionized water at a variety of conditions such as pH value, temperature, and immersion time. To calculate the swelling ratio, the vials were placed in a bath at various temperatures, and the synthesized hydrogel was separated from the vials at given times, wiped with filter paper, weighed, and placed in the same vials. The swelling ratio of the samples was calculated using the following equation:


(1) $$SR\%=\frac{W-W_{0}}{W_{0}}\times 100$$

where W is the weight of swelled hydrogel, and W_0_ is the weight of hydrogel after drying.

Also, the swelling degree was analyzed at various temperatures of 25–75 °C and pH value of 2–14. Moreover, the study of swelling behavior was carried out by various aqueous solutions of NaCl and Na_2_SO_4_ (0.001, 0.01, and 0.1 gr of salt).

Further study was performed to track the swelling properties and water diffusion of polymer networks. For this purpose, Fickian diffusion model and Schott’s 2nd-order-kinetic model are utilized [18].

## 3. Results and discussion

### 3.1. FT-IR analysis of amphiphilic hydrogel

**Figure 1:**
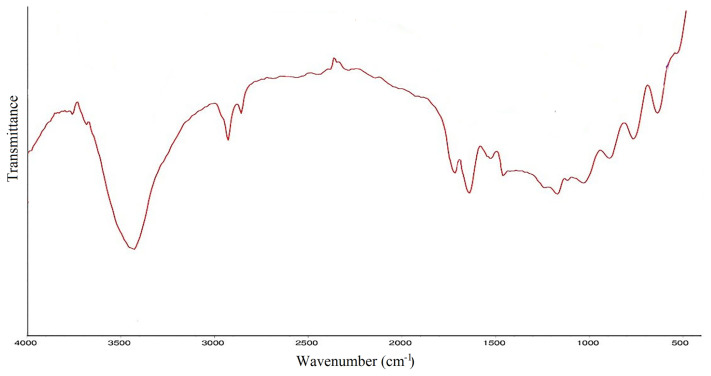
FTIR spectrum of AA/CS hydrogel.

Fourier transform infrared (FT-IR) spectroscopy was used to show the spectrum of the synthesized amphiphilic semi-IPN hydrogel. As can be seen from Figure 1, the very broadband extending from 3400 to 2400 cm^−1^ is attributed to O-H stretching band. This broadband obscures the C-H and N-H stretching bands of CS. Other characteristic peaks of AA are C=O band appearing in the range of 1730–1700 cm^−1^ and O-H out of plane bending in the range of 1000–900 cm^−1^. The peaks that appeared at about 1320 cm^−1^ and 890 cm^−1^ are attributed to C-N and pyranoid ring stretching of CS, respectively [19–20].

### 3.2 Swelling behavior of amphiphilic hydrogel

A variety of parameters such as applied environment, chemical properties, structural characteristics, etc. could influence the swelling properties of hydrogels in a given media. Taking that into account, a lot of scientific research in this field is still ongoing [20–22]. Therefore, in this study, the changes in the swelling behavior of the semi-IPN amphiphilic hydrogels have been monitored in different conditions.

**Figure 2:**
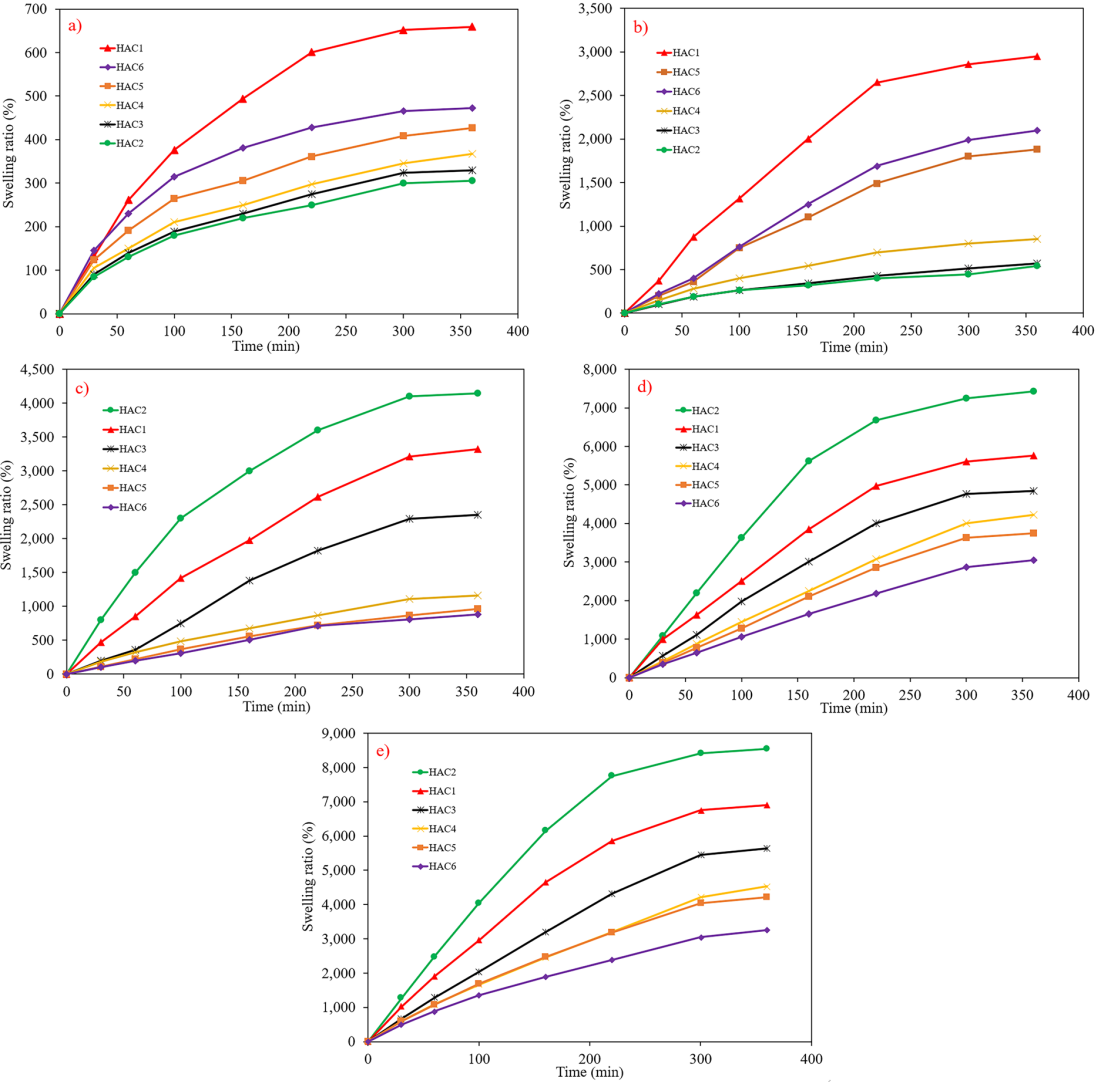
Swelling ratio percentage versus time at different pH values, a) pH = 2, b) pH = 5, c) pH = 7, d) pH = 10 and e) pH = 14.

Figures 2a–2e show the pH- and time-dependent swelling of the synthesized hydrogels having a various amount of CS. As can be seen, all the samples swelled sharply and reached equilibrium after 5 h. It is worth pointing out that through the time of the swelling-deswelling process, the pH value is principally the crucial parameter to control the swelling behavior [23,24]. At a higher pH value, it was revealed that the swelling ratio increases discernibly. Because, at lower pH values, because of the presence of H^+^, Cl^−^, OH^−^, and steric hinderance, swelling of AA acid was not significant. [25]. Moreover, by increasing the CS content, both dynamic and equilibrium swelling ratios in the solution of acidic pH proliferated. This fact can be ascribed to the ionization of amine groups, which raises the swelling ratio because of electrostatic repulsions [26]. However, at a higher pH value, an enhancement in CS amount led to a decline in swelling degree. In fact, by increasing the number of amine groups, fewer carboxyl groups could be ionized, suggesting a decrement in swelling ratio [27]. The maximum swelling ratio (8550%) was detected for sample HAC2 at pH = 14. Table 2 represents the swelling degrees of the synthesized hydrogels in a variety of pH values.

In order to investigate the mechanism of swelling, Fickian diffusion and Schott’s 2nd-order-kinetics models were utilized. In the Fickian diffusion model, type of diffusion can be described using three various modes. If the swelling behavior is dominated by Fickian diffusion, the diffusional exponent (n) is less than 0.5. In this case, the simple concentration gradient is the liability for the transport of water. However, if swelling behavior is dominated by non-Fickian diffusion, (n) is between 0.5 and 1, and both water diffusion and polymer chains relaxation is responsible for the water absorption. Finally, when the swelling process is dominated by anomalous diffusion, (n) is more than 1, and the diffusion system is controlled by the relaxation of polymer chains [18]. Table 3 represents the values of the key parameters of both models. Table 3 represents that the Fickian diffusion controls the swelling process, as well as a simple concentration gradient, is liable for water absorption.

The theoretical values of the equilibrium swelling degree (S_eq_), obtained by Schott’s 2nd-order-kinetics models, are closed to the experimental results. Consequently, the chemical structure of the repeating units of monomer in the semi-IPN hydrogels affects the swelling process [18].

### 3.3 Effect of temperature on the swelling process

**Figure 3:**
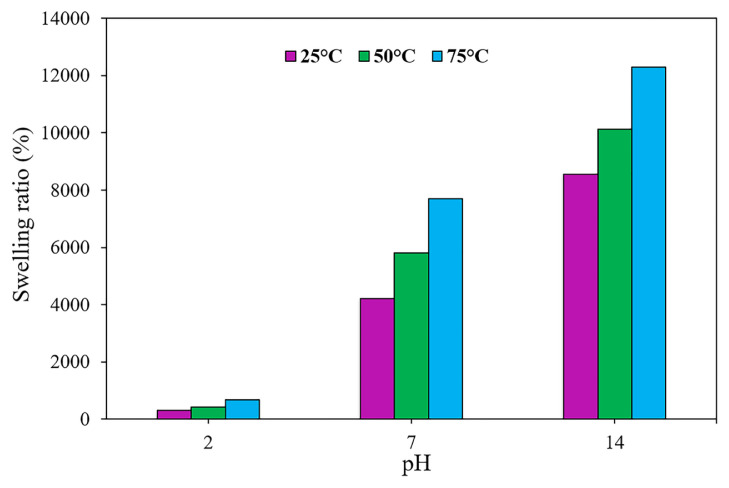
Effect of temperature on the swelling ratio in different pH values for sample HAC2.

Temperature is one of the most significant parameters in swelling behavior. It has been revealed that the Flory-Huggins interaction parameter is reliant on temperature, affecting the swelling behavior [28]. To assess the influence of temperature on the swelling process of the amphiphilic semi-IPN hydrogels, the swelling experiments were undertaken at a variety of temperatures (i.e., 25, 50, and 75 °C) and pH values. Figure 3 represents the swelling behavior of the sample HAC2 as a function of pH values at different temperatures. As can be seen, the temperature has a substantial influence on the swelling behavior of the synthesized hydrogel. For instance, at pH = 14, when the temperature enhanced to 75 °C from 25 °C, the swelling ratio grew up to 12300% from 8550%. These results reflect that by the increase in temperature, the polymer chains expand, and the thermal mobility of polymer molecules inside the hydrogel, as well as the interactions among the functional groups of hydrogels and molecules of water, enhances. Also, it should be mentioned that as temperature increases the H-bonds inside the hydrogels were broken, suggesting an increase in the swelling ratio [18, 22, 29].

### 3.4. Effect of ion strength on the swelling process

**Figure 4:**
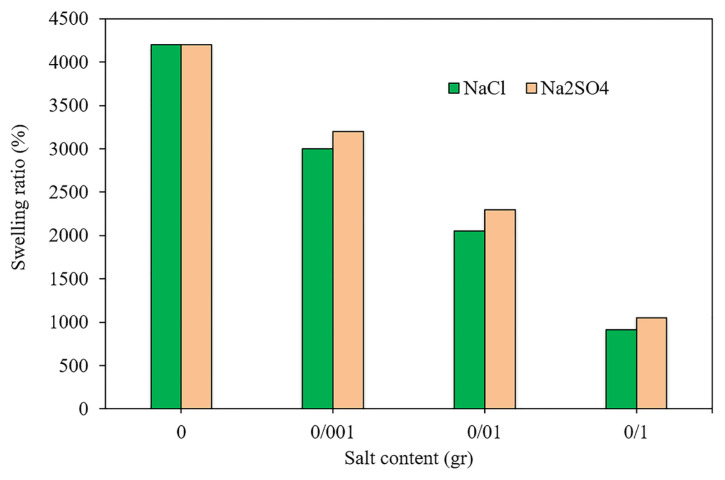
Swelling behavior of HAC2 in the aqueous solution of NaCl and Na_2_SO_4_ with different salt concentrations.

From a practical viewpoint, it is crucial to understand the swelling behavior of the novel synthesized hydrogels in different saline solutions. Thereupon, in this section, the swelling behavior of HAC2 was investigated in two different aqueous solutions (NaCl and Na_2_SO_4_) with various salt content. It is clear from Figure 4 that an improvement in the salt concentration led to an appreciable reduction in the swelling ratio of the hydrogel, because of the repulsive force of the static electricity as well as the charge balance. Also, it should be noted that the presence of counterions neutralizes the solution charges and increase the osmotic pressure of the media. This phenomenon leads to the restriction of water absorption [30,31]. Similar tendencies were revealed for other hydrogel samples and in various pH values. The results also revealed that the swelling ratio in the aqueous solution of Na_2_SO_4_ is higher than NaCl. This is attributed to that the higher number of positive and negative ions in Na_2_SO_4_, suggesting higher ionic competition in the solution [32].

## 4. Conclusion

In this study, a series of novel amphiphilic semi-IPN Acrylic acid/Chitosan hydrogels in different molar ratio in feed composition were synthesized using AIBN as a free radical initiator and N,N’methylene bis acrylamide as crosslink agent. The swelling study of the hydrogels demonstrated that the swelling degree of the synthesized hydrogels depend on the pH values and the molar ratio of the components, and the maximum swelling ratio was observed for sample HAC2 at pH = 14. It is also found that Fickian diffusion controls the swelling process. The swelling ratio was instantly correlated to the temperature and inversely correlated to the salt content of the aqueous media. These synthesized semi-IPN hydrogels are applicable in wastewater remediation such as removing the nitrophenol derivatives as hazardous waste.

## Figures and Tables

**Table 1:** The designation and composition of the synthesized semi-IPN hydrogels.

Sample	Feed composition (Mole fraction), AA:CS	NNMBA (gr)	AIBN (gr)	H_2_O (mL)
HAC1	1:0	0.0005	0.0005	1
HAC2	1:001	0.0005	0.0005	1
HAC3	1:0.005	0.0005	0.0005	1
HAC4	1:0.01	0.0005	0.0005	1
HAC5	1:0.02	0.0005	0.0005	1
HAC6	1:0.03	0.0005	0.0005	1

**Table 2:** Equilibrium swelling ratio (%) of the synthesized hydrogels in different pH values.

Sample Name	Equilibrium swelling ratio (%)				
	pH = 2	pH = 5	pH = 7	pH = 10	pH = 14
HAC1	660	2950	3410	5760	6900
HAC2	310	540	4200	7430	8550
HAC3	330	570	2460	4850	5637
HAC4	370	850	1230	4220	4530
HAC5	430	1880	964	3750	4220
HAC6	470	2100	880	3050	3250

**Table 3:** Parameters of Fickian diffusion model and Schott’s pseudo-second order kinetic model at pH = 7.

Sample Name	Fickian diffusion model		Schott’s pseudo-second order kinetic model			
	K	n	A	B	S_eq_	k_s_
HAC1	−3.1532	0.4620	0.0547	0.0004	3360	0.00929
HAC2	−2.7536	0.3850	0.0570	0.0006	4050	0.01625
HAC3	−2.6851	0.3525	0.0433	0.0003	2570	0.00819
HAC4	−2.3531	0.3349	0.0510	0.0008	1290	0.01360
HAC5	−2.7452	0.4188	0.0580	0.0004	1020	0.01323
HAC6	−2.7041	0.4035	0.0497	0.0005	740	0.01555
